# Contributing Factors to Low Energy Availability in Female Athletes: A Narrative Review of Energy Availability, Training Demands, Nutrition Barriers, Body Image, and Disordered Eating

**DOI:** 10.3390/nu14050986

**Published:** 2022-02-25

**Authors:** Andrew R. Jagim, Jennifer Fields, Meghan K. Magee, Chad M. Kerksick, Margaret T. Jones

**Affiliations:** 1 Sports Medicine, Mayo Clinic Health System, La Crosse, WI 54601, USA; 2 Exercise & Sport Science Department, University of Wisconsin-La Crosse, La Crosse, WI 54601, USA; 3 Patriot Performance Laboratory, Frank Pettrone Center for Sports Performance, Intercollegiate Athletics, George Mason University, Fairfax, VA 22030, USA; jfields2@springfieldcollege.edu (J.F.); mmagee2@gmu.edu (M.K.M.); mjones15@gmu.edu (M.T.J.); 4 Exercise Science and Athletic Training, Springfield College, Springfield, MA 01109, USA; 5 Sport, Recreation, and Tourism Management, George Mason University, Manassas, VA 22030, USA; 6 Exercise & Performance Nutrition Laboratory, Lindenwood University, St. Charles, MO 63301, USA; ckerk-sick@lindenwood.edu

**Keywords:** low energy availability, energy expenditure, energy intake, female athletes, health

## Abstract

Relative Energy Deficiency in sport is experiencing remarkable popularity of late, particularly among female athletes. This condition is underpinned by low energy availability, which is a byproduct of high energy expenditure, inadequate energy intake, or a combination of the two. Several contributing factors exist that may predispose an athlete to low energy availability, and therefore a holistic and comprehensive assessment may be required to identify the root causes. The focus of the current narrative review is to discuss the primary contributing factors as well as known risk factors for low energy availability among female athletes to help practitioners increase awareness on the topic and identify future areas of focus.

## 1. Introduction

Increased awareness, in combination with improved athlete monitoring capabilities, has resulted in a growing focus on Relative Energy Deficiency in Sport (RED-s) within the sport nutrition literature. The International Olympic Committee published a consensus statement in 2014 [1], with subsequent updates in 2018 [2], highlighting the importance of bringing awareness to RED-s, prevalence, and directions for future research. RED-s is defined as a state of impaired physiological functioning caused by (chronic) relative energy deficiency, and includes, but is not limited to, impairments of metabolic rate, menstrual function, bone health, immune function, protein synthesis, cardiovascular health, and various indices of physical performance [1,2]. A common metric used in the identification of risk factors, or diagnosis of RED-s, is the assessment of low energy availability (LEA), which is a metric used to quantify the residual energy available to support the body’s physiological functions after accounting for the energy expenditure from activity [3,4]. Historically, the Female Athlete Triad has also been an area of focus with significant overlap between RED-s [1], both of which are centered on the common themes of energy deficiency and disordered eating patterns. Since insufficient energy supply required to support athletic participation and training is a primary contributor for both RED-s and the Female Athlete Triad, LEA is viewed as a common metric of interest for both conditions. By definition, LEA is a byproduct of inadequate energy intake, high energy expenditure from high training demands, or a combination of the two. However, several underlying risk factors may be present beyond insufficient energy intake or excessive energy expenditure that predispose an athlete to LEA.

**Figure 1:**
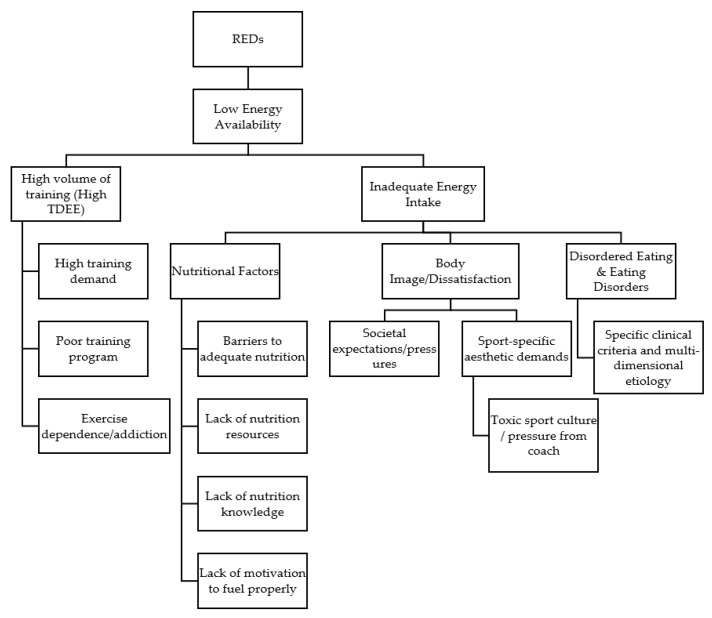
A theoretical framework outlining potential risk factors and contributors to LEA and RED-s.

The physiological effects, as well as the performance and health implications of RED-s, have been well-studied [2,3,5,6]. However, the overlapping nature of underlying contributing factors has yet to be explored completely. While a mismatch between energy intake and energy expenditure is the root cause, it is unclear why certain athletes appear to be more likely to under-fuel and ultimately predispose themselves to LEA and RED-s. Athletes may consciously or unconsciously lower their energy intake, either of which can increase the risk for LEA, particularly if this pattern continues over time. Currently, no explanations are clearly established that detail why some athletes restrict energy intake at times; whereas a number of underlying issues, risk factors, barriers, and complex etiologies are likely present that may predispose one to having LEA, RED-s, or meeting the criteria for the Female Athlete Triad. Such etiological factors may include exercise dependence, body image, disordered eating, or clinical eating disorders. Furthermore, participation in certain sports such as weight-sensitive, weight class, aesthetic, or any sport where it may be presumed that a lower body mass results in improved performance, or all of these factors, which may also exist on a spectrum and are likely to be connected through multiple constructs [1,2,3,5,7,8]. Further, certain barriers such as financial resources, access to nutritional support, and lack of time may also contribute to an athlete’s overall risk for LEA. Due to the diverse and complex nature of the etiologies that underpin the development and risks for LEA and RED-s, it is necessary to provide a multifaceted and holistic view of how these conditions can develop. Figure 1 provides an illustration of a theoretical framework outlining the potential risk factors and contributors to LEA, which will later be discussed in this review.

It is important to also consider a comprehensive view of these constructs and the spectrum of underlying risk factors that exist in order to more appropriately prevent, screen, and identify those at risk, while also being able to provide treatment when needed. It is important to note, that part of the rationale behind the development of RED-s was to better highlight the possibility that these challenges can also be experienced by males, and they are not solely exclusive to females. Indeed, studies have highlighted the presence of LEA in some male athlete populations, but there appears to be a higher prevalence among female athletes [3]. This may be partly attributed to underlying body image issues, the culture of their respective sports [9], and societal pressure to attain a certain body type or aesthetic appearance [7,10]. As such, the purpose of this narrative review was to identify known risk factors and to further discuss their contributions to LEA in an effort to recognize areas of overlap and identify directions for future research and interventions specific to female athletes. While it may be difficult to examine the relationships between all risk factors in isolation, in addition to prevalence rates, and the potential for primary causality or predictive odds of having LEA, several areas of focus can be explored and used to establish screening tools and discussion points for future educational programs. For the purpose of the current review, the following six categories will be addressed: prevalence of LEA, nutritional factors impacting dietary behaviors, training demands and energy expenditure, body image, disordered eating, and practical recommendations and future directions.

## 2. Prevalence of Low Energy Availability

A growing body of research highlights the prevalence of LEA across female athletes, participating in a wide range of sport types and levels of competition [3]. While multiple strategies exist to quantify LEA, all of them require the assessment of energy intake, body composition, and activity energy expenditure (AEE) [11]. Energy availability is calculated by subtracting AEE from daily energy intake, which is then expressed per unit of fat-free mass in kilograms [6], with <30 kcal·kg FFM^−1^·day^−1^ often used as the threshold to categorize energy availability as ‘low’ [11]. Other strategies may include the use of screening tools such as the low energy availability in females questionnaire (LEAF-Q), which is designed to identify those who may be at increased risk based upon history of symptoms [12]. While certain sports may pose a higher risk of LEA, recent evidence indicates that LEA can be an issue for athletes competing in any sport [3,13]. However, athletes participating in sports that require a high volume of training (i.e., endurance and ultra-distance sports), which subsequently results in a high total daily energy expenditure (TDEE), are likely at a greater risk for LEA, as it may be challenging to consume an adequate amount of energy to offset the high energy expenditure from training [14,15]. Prevalence of LEA among certain National Collegiate Athletic Association (NCAA) Division I athletes has ranged from 41% in cross country runners [16] to 51% in track and field athletes [17]. Further, athletes competing in team field sports with a high aerobic component (i.e., soccer), tend to also be at a higher risk, likely due to the high activity energy expenditures [3,18] associated with the sport, with prevalence of LEA ranging from 11% [19] to 67% [18], depending upon the time point throughout the season (i.e., off-season vs. pre-season vs. in-season).

Certain sports, commonly referred to as weight-sensitive sports, in which a low body fat percent, or lower body weight are often desirable, may also pose a higher risk of LEA, as athletes may intentionally restrict food intake to promote weight loss [3,13,20]. Such sports include dance, ballet, figure skating, gymnastics, synchronized swimming, as well as weight-class, and physique sports (i.e., wrestling, mixed martial arts, bodybuilding, and figure competitions). During a 7-day monitoring period Torres-McGehee et al. [20] reported that 96.2% of ballet dancers (25/26) were classified has having LEA based upon assessment of energy intake and activity energy expenditure. Additionally, Schaal et al., in 2017 [21] found that 100% of the synchronized swimmers assessed, displayed LEA, while Costa et al. [22] later in 2018 reported that 52% of synchronized swimmers displayed LEA. Moreover, in the study by Costa et al., the mean resting metabolic rate (RMR) ratio (measured RMR/predicted RMR) ranged from 0.85–0.91, which has been proposed as a risk factor for energy deficiency if the ratio is <0.90 [23].

A summary of studies examining the prevalence of LEA among female athletes is provided in Table 1. Readers are also directed to previous reviews on the topic for a more comprehensive summary of LEA [2,3,13]. While temporary energy restriction for the promotion of weight loss is common practice among weight class sports, it is currently unknown how detrimental this practice is in regard to the acute, but cyclical, time spent with LEA for these athletes. More research is needed to determine how much time spent in a LEA state is required, before it begins to elicit health perturbations and decrements in performance. It is worth noting, that a limitation of dichotomously categorizing athletes as those with or without LEA, is that it may not be applicable to all populations and sport types as individual differences may influence the magnitude of health perturbations for those with and without LEA. Moreover, a dichotomous categorization downplays the potential that there is a likely a dose-dependent relationship [24], or more of a spectrum, in regards to LEA, and the subsequent impact on health and performance. Further, time spent in a LEA state is also likely to influence the magnitude of health perturbations. Another limitation is the current discordance in the literature regarding the threshold of energy availability at which health perturbations begin to arise. As evidenced, early work by Loucks et al. [25,26] observed disruptions in luteinizing hormone pulsatility and low triiodothyronine-3 (T3) levels [27] at a threshold of energy availability, whereas Reed et al. [28] recently observed that energy availability was not associated with menstrual status across the entire spectrum of menstrual disturbances, only appearing to discriminate at clinical extremes—only to be later refuted by Loucks et al. in 2019 [29]. Lastly, recent evidence [30] indicates that low carbohydrate availability may have just as much, if not a greater, influence on health issues compared to LEA alone. Therefore, more work is warranted to elucidate these varying circumstances and confounding variables surrounding the health implications across a spectrum of energy availability values.

## 3. Nutritional Factors Impacting Dietary Behaviors

Inadequate, and potentially inadvertent, energy intake, is known to be a primary contributor to LEA among athletes [3] and can be characterized as a component of disordered eating. A key consideration for the determination of energy availability stems from the widespread underestimation of dietary intake that is known to occur in human populations, [43,44] including athletes [45,46]. Thus, researchers in this area must take prudent measures to optimize the accuracy of the dietary assessment being completed, so as to not overestimate the prevalence of LEA. In addition, athletes often underestimate the energy demands of their sport, which causes further problems with EA assessment, as well as increasing the likelihood of inadequate energy intake [47,48]. One potential explanation for this misunderstanding, may stem from confusion in regard to understanding the distinction between dietary strategies for weight loss versus dietary strategies for performance. Oftentimes, healthy eating strategies portrayed in mainstream media have an underlying theme of calorie restriction (directed towards weight loss); therefore, athletes may not understand the distinction between eating for weight loss and eating for performance. As a result, athletes may restrict energy intake under the assumption that restricting certain foods or food groups (i.e., carbohydrates) or following eating patterns, such as time-restricted eating, is considered healthy. While the importance of healthier food options cannot be understated, this can become problematic for athletes who require 2500–4000 kcal·day^−1^ to maintain energy balance. Collectively, these issues highlight a common finding that athletes have a poor level of sport nutrition knowledge. Previous research has consistently reported poor, insufficient, or inadequate levels of sport nutrition knowledge among a variety of athlete populations from varying levels of competition [48,49,50,51,52]. However, there has been a large discrepancy in nutrition knowledge scores, most likely resulting from a multitude of factors including education, level of competition, survey tool employed, and available resources (Table 2) [49,52,53]. A summary of studies that have assessed the sport nutrition knowledge of athletes can be found in Table 2. Common themes include a lack of knowledge relative to vitamins and minerals, appropriate fluid and recovery strategies, weight management, and supplement use [54,55,56,57,58,59]. Athletes generally scored higher on sections addressing the consequences of hydration/dehydration, and dietary sources of nutrients [57,58]. Additionally, previous research has indicated college athletes significantly underestimate their energy and carbohydrate requirements, based upon their level of activity [47,48]. This underestimation is likely to contribute to a higher risk of LEA, and low carbohydrate availability, as athletes may not appreciate the high energy and carbohydrate intakes recommended for their sport.

Depending upon the age of the athlete, living situation, level of competition, and available resources, several potential barriers exist that may prove challenging for athletes when attempting to adhere to sport-specific nutritional recommendations [70]. Aside from knowledge, Jagim et al. [48] reported the top barriers among a cohort of NCAA Division III athletes were financial restrictions (36%), lack of time (12%), and poor access to food options (12%). In addition, food cost is a well-recognized concern for athletes living away from home with limited income [70,71]. Such athletes are balancing sport demands (i.e., training schedule, game schedule, travel schedule) with other stressors including school, work, social obligations, and family responsibilities, which may inhibit meal and snack preparation [70]. In turn, busy schedules may limit accessibility to healthier food options and subsequently increase the likelihood of choosing convenient and affordable options such as ‘fast food’. Moreover, collegiate athletes living on campus may not have sufficient cooking spaces or appliances, further reducing options in regard to preparing food. Furthermore, athletes, coaches, and dietitians have reported limited access to nutrient-dense foods when traveling, as well as in dining halls, dormitories, and sporting facilities [70].

Previous work has indicated that access to a sports dietitian can positively influence nutritional behaviors [72,73]. Hull et al. [72] reported that when a sports dietitian was indicated as the athlete’s primary nutrition source, athletes appeared to have a greater understanding of nutrient periodization, consumed less fast food, were more likely to have school-provided boxed meals while on team trips, and were more likely to prepare their own meals [72,73]. Therefore, accessibility to a sports dietitian may result in improved nutrition knowledge and dietary habits. Unfortunately, not all athletic departments and institutions may have the necessary financial resources to employ these valuable personnel. In summary, these barriers suggest athletes may benefit from sport-specific nutrition education, financial and time management strategies, and food accessibility options.

## 4. Training Demands and Energy Expenditure

Total daily energy expenditure (TDEE) is the total amount of energy expended in one day, and is the sum of basal metabolic rate (BMR; representing 60–80% of TDEE), activity energy expenditure (15–30% of TDEE), and the thermic effect of food (10% of TDEE) [74]. The TDEE fluctuates as a result of sport training, sport season, and level of performance [38,39]. The achievement of energy balance, which occurs when intake is equal to TDEE, is important to maximize sport performance while minimizing the loss of fat free mass [75], injury risk [76], infection [77], and risk of developing LEA [13]. Similarly, to maintain an adequate level of energy availability (>40 kcal·kg FFM·day^−1^), a higher energy intake is needed as activity energy expenditure increases to compensate for the increased energy output and ensure adequate energy is available to support the body’s physiological requirements. Table 3 provides a summary of studies examining TDEE and activity energy expenditure of female athletes competing in various sports. These studies highlight that TDEE of athletes varies across sports and can range from 2300–3500 kcal·day^−1^, or 30–50 kcal·kg·day^−1^ when normalized to body mass, which is higher than a typical sedentary adult (1800–2200 kcal·day^−1^) [78]. The majority of those competing in team sports (e.g., soccer, basketball, lacrosse) appear to expend approximately 2500–3000 kcal·day^−1^ which can depend upon the type of training or competition [79], and phase of the season [38,39,80]. If resting metabolic rate is known, either through direct assessment or use of prediction equations, physical activity levels (PAL) can be calculated by dividing TDEE by the resting metabolic rate. This is a way to assess the energy demands of training and daily activities, relative to baseline metabolic requirements and also serves as a practical way to estimate daily energy requirements for athletes. As seen in Table 3, PAL values can range from 1.5 to 2.2 depending on the sport and phase of the season, with ultra-distance sports or periods of high training volume potentially elevating PAL values up to ~3. While not able to independently identify athletes at risk for LEA as energy intake is not accounted for, high (>1.7) PAL values would signify a high TDEE value, relative to the individual, and therefore would warrant focused efforts to increase energy intake. It is important to also add that fluctuations in TDEE, and subsequently PAL values, are likely to occur throughout different phases of a season [38,39], and across different types of training days [79]. Therefore, PAL values could be used in combination with other metrics to potentially screen those at risk of LEA throughout various training phases of a calendar year.

For most, matching TDEE with increased energy intakes should be attainable with a conscious effort to increase food consumption throughout the day. However, ultradistance athletes or multi-stage endurance competitions (i.e., Tour de France) may be the exception, as TDEE values have been reported to exceed 10,000 [85] and 6000 kcal·day^−1^ [91], respectively, which may prove challenging for athletes to consume a sufficient energy intake to maintain energy balance during such rigorous competitions. Regardless, a failure to increase energy intake in response to elevated activity levels is likely the primary issue for most athletes, rather than excessive TDEE values, in terms of contributors to LEA. Additionally, it is worth noting that the frequency of days spent in a negative energy balance or with LEA is likely a key component of how detrimental energy deficiencies may be on performance and health parameters; however, more research is warranted to fully elucidate these associations to support this hypothesis. It is important to realize the amount of energy expended during training and throughout the entirety of a day to facilitate the adoption of effective nutritional strategies. Sufficient fueling and nutritional periodization strategies during periods of high energy expenditure are essential for avoiding low energy states, optimizing performance, and maintaining health.

Another area of concern regarding high TDDE values for athletes is the concept of exercise dependence. Exercise dependence, also commonly referred to as exercise addiction or compulsive exercise, represents a behavioral pattern in which individuals feel constantly pressured to exercise, even at what would be considered excessive levels of activity, regardless of the potential harmful effects [92,93]. While not always the intent, exercise dependance is likely to be associated with negative energy balance and weight loss [93]. Further, exercise dependence has also been previously associated with eating disorder behaviors, which are factored in to the diagnostic criteria [94], and further highlights the need for behavioral modification therapy for those suffering from these overlapping and problematic behaviors [93]. While it may be intentional at times, exercise dependence can also contribute to increased energy expenditure and further predispose an athlete to LEA and RED-s as has been observed in males [93]; however, mixed findings have been reported in regards to a similar relationship being present in females [95,96], warranting further work in this area to explore the role of exercise dependence as a risk factor for RED-s. Similarly, athletes may also increase exercise activities, in addition to or outside of regular sport activities, in a conscious effort to increase energy expenditure with the intent to lose weight. This is a common practice among wrestlers trying to make weight for competition [97]. Again, while intentional at times, this may still represent an area of concern for athletes if they are exercising at levels considered to be excessive and beyond the regular demands of their sport. This behavior may in turn increase their risk for overtraining or compromise recovery, both of which may be further exacerbated if energy is being simultaneously restricted [98]. Together, these strategies are common weight cutting strategies employed by wrestlers [97] and other combat sport athletes [99] that warrant careful oversight.

## 5. Body Image

Historically, the term body image refers to the internalization of how one views oneself regarding their body and outward physical appearance, which can subsequently influence cognitive, emotional, and behavioral aspects [100,101]. Body image is largely influenced by society and mass media, and more recently, social media platforms [102,103]. A framework centered around body image and eating disturbances, known as the tripartite model of influence, hypothesizes that societal influences such as media, peers, and parents have a strong influence on the internalization of a ‘thin ideal’ body type for young females [104]. This framework has been associated with eating disturbances, body dissatisfaction, and low self-esteem, with a drive for thinness commonly associated with aforementioned disordered eating and body dissatisfaction that frequently co-exist in young females [104]. Body dissatisfaction occurs when individuals feel that their internal view of self-body image does not align with what they perceive as an ‘ideal body’ and is frequently associated with a drive for thinness. Therefore, body dissatisfaction is seen as a risk factor for disordered eating patterns [105,106], and is common among athletes who present with disordered eating patterns. Body dissatisfaction can often lead to an intentional restriction of food intake or excessive exercising in an effort to alter body appearance [106,107].

Despite athletes often being leaner than non-athlete populations, research has found that some athletes may compartmentalize their ‘sporting’ body and their ‘social’ body, in which they understand their body type might be more favorable when compared to non-athlete populations. However, previous work has indicated that athletes may have stricter expectations for their ‘sporting’ body and may still feel a level of dissatisfaction [7,100]. Further, influences from society and social media may leave athletes feeling pressured to conform to a certain body type standard or aesthetic appearance, regardless of the impact upon sport performance, are more increasingly present [103,108]. While body image and dissatisfaction may be an issue for both male and female athletes, previous research has primarily focused upon female athletes [9]. Further, there are several sports in which there is pressure and unwritten expectations to look a certain way or have a certain body type. Often, these expectations are centered around being small statured, petite, lean, or ‘light’ (in the context of body weight), with the belief that it may improve physical performance or appease judges if subjective assessments are integral to the sport (e.g., gymnastics, figure skating, synchronized swimming) [7]. This may partially explain why athletes who compete in leanness-focused sports tend to have higher rates of disordered eating compared to sports that do not have weight class, body shape, or body weight expectations and degrees of subjectivity [8]. Importantly, not all such cases of distorted body image are in these types of sports as a previous study completed in female rugby athletes (a strength and power, collision sports, with an emphasis on lean body mass) also reported athletes who struggle with body image [109].

Body dissatisfaction can lead athletes to believe they are overweight and need to lose weight, regardless of their weight status or body fat percentage. Previous work has identified a higher percentage of lean women, compared to men, self-report a perception of being overweight and acknowledge multiple weight loss attempts, despite already having a normal or low body mass index [110]. In a sample of NCAA DI women equestrian athletes, the athletes perceived size of their body was significantly larger than their actual body size, which was also accompanied by the desire to wear smaller clothing and uniform sizes [111]. Similarly, Jagim et al. [48] noted that 35% of the collegiate women athletes assessed, reported a desire to lose weight, despite having a collective mean body fat percentage of 24% and a mean body mass index of 23.3 kg·m^2^, which places them in the ‘fair/poor’ body composition range and ‘normal weight’ categories for women. In support of previous work [2,107], Reed et al., 2013 [19] reported a higher level of body dissatisfaction in collegiate soccer athletes with LEA, indicating that body dissatisfaction may represent a potential causative factor in regard to LEA and RED-s. Additionally, when examining the team as a whole, Reed et al., 2013 [19] reported that a negative relationship existed between energy availability, body dissatisfaction, and a drive for thinness [19].

The overlapping nature of body dissatisfaction and restrictive eating habits may predispose those dissatisfied with their body image or with a high drive for thinness to employ restrictive eating behaviors in order to achieve their desired body image [100]. The importance of screening such athletes for disordered eating cannot be overemphasized and plays an important role in the prevention and treatment of athletes with RED-s.

## 6. Disordered Eating

**Figure 2:**
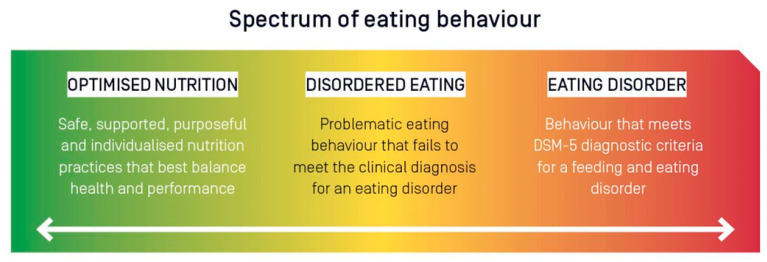
The spectrum of eating behaviour in the high performance athlete from optimised nutrition to disordered eating to eating disorders. DSM-5, Diagnostic and Statistical Manual of Mental Disorders, Fifth Edition. Figure originally published by: Wells, K.R., et al. The Australian Institute of Sport (AIS) and National Eating Disorders Collaboration position statement on disordered eating in high performance sport. Br. J. Sports Med. 2020 Nov; 54(21): 1247–1258.

To determine the appropriate course of action or necessary treatment, a distinction should be made between disordered eating and a clinical eating disorder. As seen in Figure 2, within the field of sports nutrition, disordered eating operates on a spectrum of severity, which may be signified by: (A) mismatches between energy intake and energy expenditure; (B) poor dietary habits (e.g., infrequent eating frequency, skipping meals, eating excessive amounts of fast food or ‘junk’ food); (C) poor nutrient density in the diet; (D) concerns of body weight (often leading to use of binge eating, laxatives, diuretics, diet pills and other extreme weight loss measures); or (E) any other abnormal dietary pattern [107,112].

Conversely, a clinical eating disorder is a diagnosable condition that meets the criteria established by the Diagnostic and Statistical Manual of Mental Disorders (DSM) published by the American Psychiatric Association and is an example of a more problematic pattern of eating behavior [107]. A recent consensus statement from the International Olympic Committee outlined important characteristics and traits to distinguish between the two [113]. In both scenarios, treatment often requires a multidisciplinary approach from behavioral health experts, registered dieticians, and sports medicine practitioners. Moreover, in both conditions, athletes rarely self-report symptoms, typically hiding the behaviors until they become problematic, which oftentimes leads to more severe health outcomes [107]. Further, LEA and RED-s can occur with or without a clinically diagnosed eating disorder [114]; therefore, any athlete with a disordered eating pattern should be considered at risk for RED-s.

**Figure 3:**
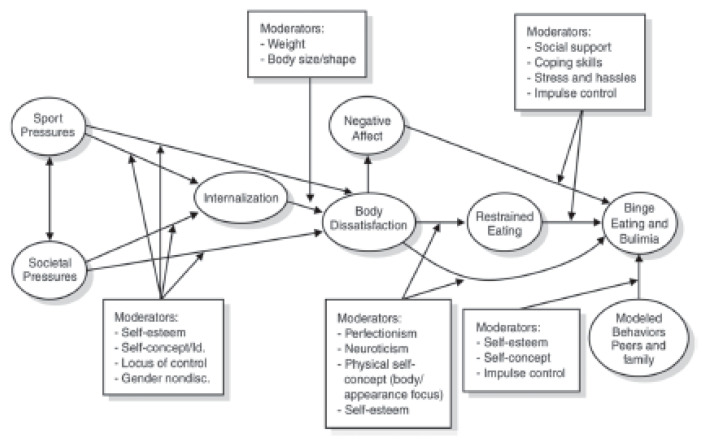
Etiological model showing the interplay of eight risk or causal risk constructs (shown in circles) considered as factors in the development of disordered eating in athletes (Petrie & Greenleaf, 2012). Used with permission from license # 5232220735165, granted on 18 January 2022. Cited within Stoyel, H., Slee, A., Meyer, C., Serpell, L. Systematic review of risk factors for eating psychopathology in athletes: A critique of an etiological model. Eur. Eat Disord. Rev. 2020 Jan; 28(1): 3–25.

Estimates indicate that the overall prevalence of disordered eating and/or eating disorders among athletes ranges from 0 to 19% in men and 6 to 45% in women [107], both of which are higher than non-athletes, which is estimated to be 0.7–2.2% and 2.2–8.4% for adult men and women, respectively [115]. Previous literature identified ~25% of NCAA DI collegiate athletes from a variety of sports including gymnastics, softball, synchronized swimming, tennis, basketball, lacrosse, soccer, cross country, cheerleading, diving, field hockey, swimming, and track and field to exhibit disordered eating behaviors [116]. Further, several studies have examined the relationship between disordered eating and sport type, specifically the differences between lean sports, where an emphasis is placed upon low body weight and physique, versus non-lean or ball sports [117,118,119,120,121,122]. Underlying risk factors that were identified with the onset of eating disorders include participation in weight-sensitive sports with a pressure to maintain a lower body weight or leaner physique [107]. Previous literature has also reported wrestlers and gymnasts have a greater drive for thinness as well as a higher incidence of food restriction and purging behaviors compared to other sports [123]. However, athletes of any body size and composition can exhibit disordered eating patterns. Arthur-Cameselle and Quatromoni [124] identified internal factors such as *Negative Mood States, Low Self Esteem, Perfectionism/Drive for Achievement*, and *Desire for Control* and external factors including *Negative Influences on Self-Esteem*, *Hurtful Relationships, Hurtful Role Models, and Sport Performance* to be associated with eating disorders in women collegiate athletes. Ravi et al. [125] identified that a prior history of eating disorders was associated with menstrual dysfunction and injury rate, which are known health issues associated with RED-s. While there are several known risk factors associated with the development of both disordered eating patterns and clinically diagnosed eating disorders, there continues to be a need for more work in this area, particularly among athlete populations to develop more efficient screening tools and educational interventions in an effort to reduce the severity and associated health consequences when these issues are not addressed. Figure 3 provides an illustration of an etiological model demonstrating the potential interplay between eight risk or causal risk constructs considered as factors in the development of disordered eating in athletes.

Another common behavioral pattern that may play a role with disordered eating in athletes is orthorexia nervosa, which is described as a pathological fixation on healthy eating [126]. This condition may predispose athletes to feel pressured to eat healthy foods, which may lead to a compulsive pattern of eating and lead to restrictive eating, malnourishment, nutrient deficiencies, in addition to isolation and anxiety regarding the more social aspects of eating. While more commonly reported in fitness enthusiast and gym attendees, previous work has identified athletes and sport participation to be risk factors for orthorexia nervosa [127]. While recognized as a problematic behavioral eating pattern, orthorexia nervosa does not currently meet the DSM criteria for a clinical eating disorder. However, it is still important for practitioners to be aware of the warning signs and provide support to athletes who exhibit the problematic eating patterns associated with this condition.

Athletes with access to a registered dietitian tend to display better eating behaviors than those without access. Further, athletes without access to a registered dietitian are more likely to seek nutrition information from a coach, [52] which could be problematic depending upon the coach’s level of sport nutrition knowledge [59]. For example, when coaches lack proper nutrition knowledge and an understanding of the risks associated with LEA, they may inappropriately instruct athletes to lose weight or follow feeding approaches that do not adequately deliver the energy and nutrients for optimal health and performance. Moreover, an established culture of emphasizing and creating pressure to lose weight can create an unhealthy, toxic culture that has been reported to elicit disordered eating behaviors, body dissatisfaction, and result in eating disorders [7,128,129]. Therefore, it is recommended that athletes have access to a sports dietitian, but if not feasible, then the design and implementation of sports nutrition education programs for coaches and athletes is suggested.

## 7. Practical Recommendations and Future Directions

In conclusion, there are areas in need of further research to address LEA and RED-s in female athletes. The underlying cause of LEA is likely multifactorial as several of the potential risk factors have been discussed in the current review. Recognizing the signs of disordered eating and clinical eating disorders is imperative for identification of those at risk of RED-s. However, it is worth noting that even in the absence of LEA, inadequate energy intake or a misalignment of dietary habits with sport-specific nutritional recommendations may also result in performance or health decrements. Additionally, the aforementioned psycho-social behaviors should also be monitored to ensure a healthy body image and subsequent relationship with food. Further, examining the root cause of any disordered eating patterns using a holistic screening process may help to improve the issue and reduce the likelihood of future problems. While LEA is one of the more common metrics used to identify those at risk for energy deficiency, other factors such as poor nutrition knowledge, high TDEEs, disordered eating behavior patterns, and body dissatisfaction can also be used to identify those at risk as they are also likely to increase the likelihood of LEA, both independently and concomitantly, ultimately predisposing athletes to RED-s. Further, a limitation of only using LEA, particularly when using as a dichotomous categorical-based metric, is that it may overlook other contextual factors and problematic behaviors that may also warrant attention. Therefore, as mentioned earlier, completing a comprehensive screening of an athlete to consider a multitude of potential risk factors is necessary. A summary of commonly used screening tools for LEA, eating disorders, body dissatisfaction, and female athlete triad is provided in Table 4.

**Figure 4:**
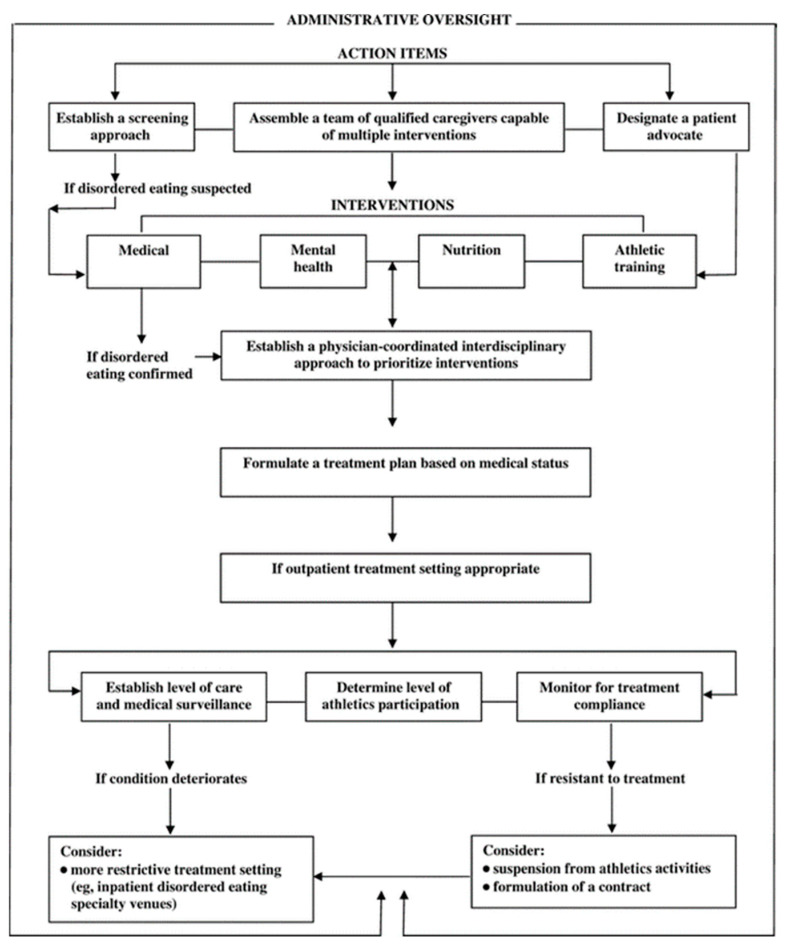
Disordered-eating management protocol: outpatient setting. Figure originally published by: Bonci, C.M., Bonci, L.J., Granger, L.R., Johnson, C.L., Malina, R.M., Milne, L.W., Ryan, R.R., Vanderbunt, E.M. National Athletic Trainers’ Association Position Statement: Preventing, Detecting, and Managing Disordered Eating in Athletes. J. Athl. Train 2008 Jan–Feb; 43(1): 80–108.

While the obvious strategy for correcting LEA is to focus on increasing energy intake or reducing activity energy expenditure [150], as discussed in the current review, underlying issues such as disordered eating tendencies or body image concerns may contribute to the issue of LEA and therefore warrant attention. As such, early educational interventions will likely be instrumental in helping improve any behavioral tendencies (e.g., energy restriction, exercise dependance, etc.) that predispose athletes to LEA. Fortunately, several of the health perturbations associated with RED-s, such as menstrual dysfunction or bone health (i.e., reductions in bone mineral density or history of stress fractures), can be improved upon over time, if proper corrective actions are taken [1,150]. Practitioners should refer to the RED-s Risk Assessment Model and the Triad return to play model in making an informed decision regarding sport participation and return-to-play for diagnosed athletes [1,2,151,152]. Additionally, Figure 4 provides an illustration of a working model for a disordered eating management protocol for outpatient settings that outlines action items, potential interventions, and key personnel required to address this issue in athletes.

While difficult to ascertain in research settings, it cannot be overlooked that some athletes may underestimate the value of optimal sport nutrition practices and, therefore, may not prioritize optimal fueling strategies. Anecdotally, several athletes may simply choose not to adhere to recommended dietary strategies and skip meals or peri-workout feeding opportunities as they choose not to prioritize these strategies or do not understand their importance. These issues are likely exacerbated when athletes do not have adequate nutritional support services or access to sport dieticians or other knowledgeable professionals who can provide sound guidance [72,73]. It is recommended future research focus on the development and employment of educational interventions to help athletes understand the specific nutritional requirements of their sport and the detrimental health and performance outcomes of under-fueling. Embracing the requirement of a high energy intake and having a physique or bodyweight that enables optimal performance and health should be a primary goal for every athlete regardless of societal expectations.

While energy deficiency continues to be a focus of sport nutrition-related research, it is clear that more work is needed in this area. Below is a list of potential areas needing to be further explored in this area:

1. The development of more efficient screening tools to identify those at risk of RED-s and the female (and male) athlete triad, particularly those encompassing the multi-factorial nature of underlying risk factors.
2. Develop a better understanding of the specific health and performance implications of energy deficiencies across a spectrum of energy availability ranges:
  - Metabolic dysfunction;
  - Hormonal disturbances;
  - Menstrual dysfunction;
  - Bone health;
  - Immune function;
  - Performance implications.
3. Evaluate time-dependent effects of varying ranges of energy availability values to examine how long of a state of energy deficiency can be maintained prior to the onset of health and performance decrements.
  - Assess the efficacy of refeeding strategies for athletes competing in weight-class or aesthetic based sports where a state of energy deficiency is often required to lose weight or reduce body fat.
4. Evaluate the efficacy of various educational-based interventions to educate athletes on the importance of fueling strategies to support their performance and health.

## Figures and Tables

**Table 1:** Prevalence of low energy availability (LEA) in female athletes.

Author Year	Athlete Population	Duration	LEA (kcal·kg FFM^−1^·Day^−1^) Mean ± SD	% Athletes with LEA	Implications of LEA
Soccer					
Magee 2020 [18]	NCAA DIII soccer (*n* = 18, height: 1.67 ± 0.05 m; body mass: 65.3 ± 7.9 kg; body fat %: 24.9 ± 5.6%)	4 days	All: 27.5 ± 8.9 LEA: 23.0 ± 5.7 Non-LEA: 36.4 ± 7.3	The screening tool classified 56.3% of athletes as at risk of LEA. Actual dietary intake identified 67% as LEA.	N/A
Morehen 2021 [31]	Professional soccer (*n* = 24; height: 168.1 ± 5.9 cm; weight: 62.1 ± 4.7 kg; body fat%: 20.6 ± 3.7%	9-day international training camp (4 training days, 1 rest day, 2 travel days, 2 match days)	(*n* = 17) 18 ± 9 (range: 2–36)	<30 kcal·kg FFM^−1^·day^−1^, 88% of players	N/A
Cherian 2019 [32]	Junior soccer (*n* = 19; age: 12.2 ± 1.83 years; height: 1.54 ± 0.04 m; weight: 45.1 ± 6.58 kg; body fat%: 23.8 ± 3.46%)	3-days	All: 27.1 ± 14.44 U12: 31.7 ± 10.10 U16: 24.1 ± 12.32	<30 kcal·kg FFM^−1^·day^−1^, 58% of girls, of which 37% were Under-16 players.	N/A
Moss 2020 [33]	Professional soccer (*n* = 13) (age: 23.7 ± 3.4 yrs., height: 1.69 ± 0.08 m, body mass: 63.7 ± 7.0 kg)	5 days during a competitive season	All Days: 35 ± 10 Rest Days: 42 ± 7 Light Days: 35 ± 11 Heavy Day: 29 ± 10 Match Day: 29 ± 16	30–45 kcal·kg FFM^−1^·day^−1^: 62% LEA (<30 kcal·kg FFM^−1^·day^−1^): 23%	LEA athletes met criteria for low resting metabolic rate. Other biochemical markers were inconclusive.
Reed 2013 [19]	NCAA DI soccer (*n* = 19, age: 19.23 ± 0.3 yrs.; height: 1.66 ± 0.0 m; weight: 60.6 ± 1.4 kg; body fat%: 22.5 ± 1.1% VO_2_ Max: 57.0 ± 1.0 mL kg(^−1^) min(^−1^))	3-day monitoring at Pre-, Mid-, and Post-season time points	Mid-season: 35.2 ± 3.7 Post-season: 44.5 ± 3.7 (*p* = 0.009)	Low energy availability (<30 kcal·kg FFM^−1^·day^−1^) was observed: Pre: 5/19 (26.3%) Mid: 5/15 (33.3%) Post: 2/17 (11.8%)	N/A
Track & Field and endurance athletes					
Heikura 2018 [34]	National/world-class distance runners (*n* = 25; age: 23–27 years; height: 1.69–1.83 m; body mass: 52.9–70.5 kg)	7-day monitoring	N/A	LEA: 11/35 (31%)	Amenorrheic and low testosterone athletes had significantly lower sex hormones, triiodothyronine, and bone mineral density, with a ~4.5-fold increased prevalence of bone injuries.
Beerman 2020 [16]	NCAA DI cross country (*n* = 20, age: 20.2 ± 1.7 years; height: 1.77 ± 0.06 m; body mass: 53.7 ± 6.5 kg; body fat %: 23.3 ± 3.6%)	3-month average	32.8 ± 16.1	30–44 kcal·kg FFM^−1^·day^−1^: 7 (41%) <30 kcal·kg FFM^−1^·day^−1^: 7 (41%)	N/A
Day 2015 [17]	NCAA DI track and field (*n* = 25, age: 19.5 ± 1.8 years; height: 1.69 ± 0.05 m; body mass: 61.1 ± 6.9 kg; body fat %: 22.3 ± 3.3%)	3-day monitoring	30.8	<45 kcal·kg FFM^−1^·day^−1^: 23 (92%) <30 kcal·kg FFM^−1^·day^−1^: 13 (52%)	N/A
Melin 2014 [35]	Endurance athletes (*n* = 45; age: 26.6 ± 5.4; height: 1.6 9 ± 0.0; weight: 58.7 ± 6.8 kg; body fat %: 20.2 ± 3.4	7 consecutive days	All: 38.5 ± 13.9 At risk for LEA: 37.3 ± 13.1 Not at risk: 40.4 ± 15.3	At Risk for LEA (LEAF-Q>8): 28/45 (62%)	N/A
Schaal 2021 [36]	Healthy distance runners: well-adapted [WA] (age: 29.4 ± 1.6 yrs.; height: 1.65 ± 0.2 m; weight: 57.6 ± 1.6 kg; body fat %: 22.5 ± 1.4) and non-functional overreaching (NFOR) (age: 27.7 ± 2.3 yrs.; height: 1.69 ± 0.2 m; weight: 59.1 ± 3.0 kg; body fat %: 23.5 ± 1.3)	Baseline: 24–35 Days Training Overload: 4 weeks Recovery Phase: 2 weeks	Baseline: WA:24.4 ± 3.7 NFOR: 30.4 ± 1.9 Training Overload: WA: 26.3 ± 3.8 NFOR: 24.8 ± 2.8 Recovery Phase: WA: 24.3 ± 4.0 NFOR: 26.8 ± 1.6	N/A	Suppressed ovarian function. Decreased running performance.
Viner 2015 [37]	Competitive cyclists (*n* = 4; age: 38.4 ± 10.3 yrs.; height: 1.65 ± 0.06 m; weight: 62.8 ± 12.2 kg; body fat %: 24.9 ± 8.4%)	3 days·month^–1^, through one cycling season. Records were completed on alternating days each month to represent all days of the week.	Pre-Season: 26.2 ± 14.1 Competition: 25.5 ± 3.1 Off-Season: 23.8 ± 8.9	Low energy availability (<30 kcal·kg FFM^−1^·day^−1^): 100% at all time points.	N/A
Other Sports					
Zabriskie 2019 [38]	NCAA DII lacrosse (*n* = 20, age: 20.4 ± 1.8 years; height: 1.68 ± 0.06 m; body mass: 68.8 ± 8.9 kg; body fat %: 27.9 ± 3.0%)	5 periods, of 4-day monitoring	Off-season: 30.4 ± 11.0 Off-season: 26.2 ± 10.5 Pre-season: 22.9 ± 8.5 In-season: 28.7 ± 9.5 In-season: 28.9 ± 9.2	Off-season: 10/20 (50%) Off-season: 12/20 (60%) Pre-season: 15/20 (75%) In-season: 12/20 (60%) In-season: 12/20 (60%)	Associated with reduced sleep quality and perceived rest.
Zanders 2021 [39]	NCAA DII basketball (*n* = 13; age: 19.8 ± 1.3 yrs.; height: 1.74 ± 0.1 m; weight: 74.6 ± 9.1 kg; body fat %: 27.1 ± 3.2%)	5 periods, of 4-day monitoring	In-Season (non-conf): 21.8 ± 7.8 In-Season (conf): 22.3 ± 13.7 In-Season (playoffs): 22.5 ± 11.2 Off-Season I: 31.8 ± 8.1 Off-Season II: 30.6 ± 9.5	In-Season (non-conf): 10/11 (91%) In-Season (conf): 10/11 (91%) In-Season (playoffs): 5/9 (56%) Off-Season I: 3/10 (30%) Off-season II: 6/11 (55%)	N/A
Braun 2018 [40]	Elite soccer (*n* = 56; age: 14.8 ± 0.7 yrs.; height: 166 ± 5 m; weight: 56.8 ± 6.1 kg; body fat%: 17.2 ± 3.9%)	7-day food & activity records	30.0 ± 7.3 Range: 20.3 to 51.0	LEA (i.e., <30 kcal·kg FFM^−1^·day^−1^): 53%	N/A
Woodruff 2013 [41]	University volleyball (*n* = 10; age: 20.9 ± 1.4 yrs.; height: 1.77 ± 0.05 m; weight: 75.0 ± 9.7 kg; body fat %: 25.2 ± 6.9%)	7-day food & activity records	42.5	LEA (i.e., <30 kcal·kg FFM^−1^·day^−1^): 2/10 (2%)	N/A
Schaal 2017 [21]	Synchronized swimming (*n* = 11; age: 20.4 ± 0.4 yrs.; weight: 58.9 ± 1.8 kg; body fat%: 17.3 ± 0.6%)	4-day food & activity monitoring period	Baseline: 25.0 ± 3.2 Week 2: 22.3 ± 1.9 Week4: 18.0 ± 2.8	LEA (<30 kcal·kg FFM^−1^·day^−1^): 11/11 (100%)	Associated with perceived fatigue and endocrine signs of conservation (i.e., increase ghrelin and decrease in leptin).
Costa 2018 [22]	Collegiate female synchronized swimmers (*n* = 21, 20.4 ± 1.6 yrs.; height: 168 ± 4.9 cm; weight: 64.4 ± 8.7 kg; body fat%: 28.4 ± 4.5% fat)	4-day food & activity monitoring. AEE was estimated using MET values	Low AEE estimate: 30.27 ± 12.6 kcal/kg FFM High AEE estimated: 26.1 ± 12.4 kcal/kg FFM	52% (11/21) were below 30 kcal·kg FFM^−1^·day^−1^ while an additional 38% (8/21) were between 30–45 kcal·kg FFM^−1^·day^−1^	N/A
Civil 2018 [42]	Vocational ballet students (*n* = 20; age: 18.1 ± 1.1 years; body mass index: 19.0 ± 1.6 kg·m^2^; body fat: 22.8 ± 3.4%)	7 days, including 5 weekdays (with dance training) and 2 weekend days (without scheduled dance training)	Weekdays 38 ± 13 Weekend days 44 ± 13 (*p* = 0.110).	Reduced energy availability (30–45 kcal·kg FFM·day^−1^: 44% LEA (i.e., <30 kcal·kg FFM^−1^·day^−1^): 22%	Association with menstrual dysfunction.
Torres-McGehee 2021 [20]	Collegiate athletes and performing artists (*n* = 121; age: 19.8 6 ± 2.0 yrs.; height: 168.9 ± 7.7 cm, body mass: 63.6 ± 9.3 kg); equestrian (*n* = 28), soccer (*n* = 20), beach volleyball (*n* = 18), softball (*n* = 17), volleyball (*n* = 12), and ballet (*n* = 26)	7 consecutive days	All: 19.5 ± 16.1 Equestrian: 21.9 ± 9.9 Volleyball: 18.6 ± 10.9 Softball: 7.8 ± 6.4 Beach Volleyball: 12.44 ± 9.6	All: 81% (96/121) Equestrian: 82.1% (23/28) Volleyball: 83.3% (10/12) Softball: 100% (17/17) Beach Volleyball: 94.4% (17/18) Ballet: 96.2% (25/26) Soccer: 30% (6/20)	N/A

LEA = Low energy availability defined as: LEA = EA <30 kcal/kg of FFM; Non-conf = Non-conference play; Conf = Conference play; WA = Well adapted; NFOR: Non-functional overreaching; N/A = Not available; AEE = Activity energy expenditure; MET = Metabolic equivalent.

**Table 2:** Summary of sport nutrition knowledge of female athletes.

Author Year	Athlete Population	Primary Variables	Results (% of Questions Answered Correctly)
Abood 2004 [60]	Collegiate soccer and basketball (*n* = 30; height: 167.4 ± 6.1 cm; body mass: 61.9 ± 5.9 kg)	42-item true/false questionnaire related to total calories, carbohydrate, fat, protein, calcium iron, and zinc	67–70%
Andrews 2016 [61]	NCAA DI (*n* = 47)	20-item questionnaire related to macronutrients, micronutrients, supplements, weight management, eating disorders, and hydration	56.5%
Cupisti 2002 [62]	Elite national adolescent (*n* = 60; height: 167.0 ± 6.0 cm; body mass: 55.8 ± 9.0 kg)	20-item questionnaire on fats, carbohydrates, proteins, vitamins, minerals, and fiber	77.6%
Dunn 2007 [63]	NCAA DI (*n* = 98)	Nutrition and Knowledge Questionnaire [9]	51.49 ± 13.57%
Grete 2011 [64]	NCAA softball (*n* = 185)	80-item questionnaire that ranged in topic from general nutrition to specific effects of nutrients	45.7 ± 4.7%
Jagim 2021 [48]	NCAA Division III (*n* = 42, height: 169.9 ± 6.9 cm; body mass: 67.1 ± 8.6 kg; fat-free mass: 51.3 ± 6.6 kg; body fat per cent: 24.2 ± 5.3%)	Abridged Sports Nutrition Knowledge Questionnaire [65]	47.03 ± 11.04%
Condo 2019 [54]	Australian rules football (*n* = 30) (age: 24.15 ± 4.1 yrs.; weight: 64.5 kg ± 8.0; height: 168.2 cm ± 7.6)	Sports Nutrition Knowledge Questionnaire (SNKQ) [66]	Median (IQR), % correct General Nutrition Concepts (46): 28 (7), 60.8% Fluid (9): 6 (7), 66.7% Recovery (7): 4 (3), 57.1% Weight Control (15): 7 (3), 46.7% Supplements (11): 2 (3), 18.2% Total Nutrition Knowledge (88): 48 (12), 54.5%
Jessri 2010 [55]	International collegiate (*n* = 98)	88-item nutrition knowledge questionnaire on nutrient type (*n* = 46), recovery (*n* = 7), fluids (*n* = 9), weight control (*n* = 15) and supplements (*n* = 11)	Nutrient type: 42.6% ± 18.6% Recovery: 38.4 ± 15.2% Fluids: 38.2 ± 17.5% Weight control: 39.1 ± 16.7% Supplements: 35.3 ± 15.4%
Manore 2017 [51]	High school (*n* = 297)	40-item questionnaire on dietary and hydration practices, attitudes towards nutrition and hydration, nutrition knowledge, and sources of nutritional information	All: 45.1% White: 48.4% Latino: 38.8%
Nikolaidis 2014 [67]	Semiprofessional soccer (*n* = 185; height: 177.5 ± 6.4 cm; body mass: 72.3 ± 8.4kg)	11-item nutrition knowledge questionnaire	5.4 ± 1.7
Rash 2008 [56]	NCAA DI track (*n* = 52)	Questionnaire related to carbohydrates, protein, vitamins and minerals, vitamin C, and vitamin E	All: 57.8 ± 1.8% Carbohydrates: 74.5 ± 17.3% Protein: 54.2 ± 16.0% Vitamins and Minerals: 62.5% Vitamin C: 33.7 ± 36.7% Vitamin E: 47.1 ± 33.8%
Rosenbloom 2002 [57]	NCAA DI (*n* = 91)	11-item questionnaire related to macronutrients, hydration, and micronutrients	Average knowledge score was 5.7 ± 1.9 (out of 11) 54% knew that carbohydrate and fat are the main energy source for activity 75% knew that eating carbohydrates would not make them fat 49% knew that high-fat meals should not be eaten 2–3 h before an event 71% believed that sugar before an event would adversely affect performance 92% knew that dehydration negatively impacts performance 95% knew that fluids should be replaced pre-, during, and post-training 80% knew that thirst is not an indicator of fluid need 53% believed vitamin and mineral supplements increase energy
Sedek 2014 [68]	Pakistani University (*n* = 50; height: 160 ± 10cm; body mass: 53.1 ± 8.6 kg)	29-item questionnaire Nutrition knowledge was classified as very good (85–100%), good (70–84%), moderate (55–69%), and weak (<55%)	57% classified as having a “good” understanding of nutrition knowledge 43% classified as having a “very good” understanding of nutrition knowledge
Shifflett 2002 [69]	NCAA D I, II and III (*n* = 52)	20-item questionnaire related to information of perceived understanding of nutritional needs, importance of healthy diet, quality of eating habits, and sources of nutrition information	52.5%
Spronk 2015 [58]	Elite (*n* = 64)	General Nutrition Knowledge Questionnaire	Total: 59.5% Sources of Nutrients: 66.2% Choosing Foods: 61.0% Diet-disease Relationships: 43%
Torres-McGehee 2012 [59]	NCAA DI, II, and III (*n* = 111)	20-item questionnaire related to micronutrients, macronutrients, supplements, weight management, eating disorders, and hydration	All: 54.9 ± 13.5% Micronutrient and Macronutrient: 51.8 ± 20.5% Dietary Supplements: 66.3 ± 19.9% Weight Management and Eating Disorders: 47.0 ± 21.9% Hydration: 54.7 ± 24.2%

ASNKQ = Abridged Sport Nutrition Knowledge Questionnaire; SNKQ = Sport nutrition knowledge questionnaire; IQR = Interquartile range; NCAA = National Collegiate Athletics Association; DI = Division I; DII = Division II; DIII = Division III.

**Table 3:** Summary of activity and total daily energy expenditures of female athletes.

Author Year	Athlete Population	Duration	Activity Energy Expenditure	Total Daily Energy Expenditure	Physical Activity Level (PAL)
Endurance Sports					
Day 2015 [17]	NCAA DI track and field (*n* = 27; height: 168.8 ± 4.7 cm; body mass: 61.1 ± 6.9 kg; body fat %: 22.3 ± 3.3%)	3 consecutive days at 2 different time points (6 days total)	711 ± 524 kcal	N/A	N/A
Edwards 1993 [81]	Elite distance runners (*n* = 9; body mass: 55.29 ± 6.18; height: 169.1 ± 5.5 cm; BMI: 19.32 ± 1.67 kg·m^−2^; body fat %: 13.0 ± 3.2%)	7-day monitoring period	N/A	2990 ± 415 kcal·day^−1^	N/A
Loftin 2007 [82]	Recreational marathon runners (*n* = 10; age: 43 ± 12 yr.; body mass: 60.8 ± 5.7 kg; height: 162 ± 13 cm; body fat: 24.9 ± 5.5%)	Indirect open-circuit calorimetry to estimate EE of recent marathon performance	2436 ± 297 kcal	N/A	N/A
Schulz 1992 [83]	Elite distance runners (*n* = 9; age: 26 ± 3 yr.; 53 ± 4 kg, 12 ± 3% body fat, and VO_2_ max: 66 ± 4 mL·kg^−1^·min^−^^l^)	6-day monitoring period training mileage (10 ± 3 miles/day).	1087 ± 244 kcal	2826 ± 312 kcal·day^−1^	1.99 ± 0.3
Trappe 1997 [84]	Swimmers (*n* = 5; age, 19 ± 1 yr.; height: 178.3 ± 2.2 cm; body mass: 65.4 ± 1.6 kg)	5-day high volume training period (17.5 ± 1.0 km.d^−1^)	N/A	5593 ± 495 kcal·day^−1^	3.0 ± 0.2
Ultra-distance					
Costa 2014 [85]	Ultra-endurance runners (*n* = 25; age: 39 ± 7 yr.; body mass: 78 ± 11 kg; height: 177 ± 8 cm)	24 h ultra-marathon (distance range: 122–208 km)		10,755 ± 1912 kcal (equivalent to 454 kcal/h)	
Soccer					
Moss 2020 [33]	Professional soccer (*n* = 13; height: 1.69 ± 0.08 m, body mass: 63.7 ± 7.0 kg)	5-day monitoring period	Rest days: 15 ± 54 kcal Light training days: 299 ± 78 kcal Heavy training days: 786 ± 159 kcal Match days: 881 ± 473 kcal	N/A	N/A
Morehen 2021 [31]	Professional soccer (*n* = 24; height: 168.1 ± 5.9 cm; weight: 62.1 ± 4.7 kg; body fat%: 20.6 ± 3.7%	9-day international training camp in (4 training days, 1 rest day, 2 travel days, 2 match days)	1058 ± 352 kcal·day^−1^ (range: 155–1549 kcal·day^−1^)	2693 ± 432 kcal·day^−1^ (range: 2105–3507 kcal·day^−1^) 43 ± 6 kcal·kg·day^−1^ (range: 33–55 kcal·kg·day^−1^) 54 ± 6 kcal·kg·day^−1^ FFM (range: 45–68 kcal·kg·day^−1^ FFM).	1.79 ± 0.24 (range: 1.4–2.2)
Mara 2015 [80]	Elite soccer (*n* = 8; height: 172.9 ± 5.5 cm; body mass: 65.1 ± 5.9 kg; body fat %: 23.2 ± 6.2%)	7-day monitoring period	Friendly game: 644 ± 72 kcal Training session: 607 ± 76 kcal	Game days: 2925 ± 144 kcal·day^−1^ Training days: 2794 ± 65 kcal·day^−1^ Rest days: 2274 ± 88 kcal·day^−1^	N/A
Yli-Piipari 2019	Division I collegiate (age: 19.86 ± 1.35 yr.) (*n* = 18: 5 tennis, 13 soccer)	4-day monitoring period (1 game/ match, 2 training sessions, and 1 rest day)		Game/Match days: 2848 ± 304 kcal·day^−1^ Training days: 2622 ± 248 kcal·day^−1^ Rest days: 1833 ± 959 kcal·day^−1^	
Reed 2013 [19]	NCAA DI soccer (*n* = 19; height: 165.6 ± 1.2 cm; body mass: 60.6 ± 1.4 kg; body fat %: 22.5 ± 1.1%)	3 consecutive days at 3 different time points (9 days total)	Pre-season: 819 ± 57 kcal Mid-season: 642 ± 26 kcal Post-season: 159 ± 28 kcal	N/A	N/A
Lacrosse and basketball					
Zabriskie 2019 [38]	NCAA DII lacrosse (*n* = 20; height: 168.5 ± 6.6 cm; body mass: 68.8 ± 8.9 kg; body fat %: 27.9 ± 3.0%)	4 consecutive days at 3 different time points (20 days total)	Off-season: 842 ± 267 kcal Off-season: 804 ± 244 kcal Pre-season: 1001 ± 267 kcal In-season: 749 ± 161 kcal In-season: 817 ± 235 kcal	Off-season: 2608 ± 378 kcal·day^−1^ Off-season: 2579 ± 376 kcal·day^−1^ Pre-season: 2798 ± 391 kcal·day^−1^ In-season: 2513 ± 248 kcal·day^−1^ In-season: 2582 ± 303 kcal·day^−1^	Off-season: 1.75 ± 0.19 Off-season: 1.72 ± 0.14 Pre-season: 1.87 ± 0.15 In-season: 1.69 ± 0.15 In-season: 1.73 ± 0.18
Kumahara 2020 [86]	Japanese collegiate lacrosse (*n* = 17; age: 20 ± 1 yr.; height: 159.0 ± 5.7 cm; body mass: 53.0 ± 5.3 kg; body mass index: 20.9 ± 1.7 kg·m^−2^	1-week period during: Preparatory [P-phase] (approximately 9-week period) Transition [T-phase] (approximately 2-week period)	P-phase 410 ± 144 kcal T-phase: 3 ± 12 kcal	P-phase 2168 ± 248 kcal·day^−1^ T-phase: 1744 ± 138 kcal·day^−1^	
Moon 2021 [79]	NCAA DII basketball (*n* = 13; height: 173 ± 13.6 cm; body mass: 74.6 ± 9.1 kg; body fat %: 27.1 ± 3.2%) and lacrosse (*n* = 20; height: 168.4 ± 6.6 cm; body mass: 68.8 ± 8.9 kg; body fat %: 27.9 ± 3.0%)	4 consecutive days at 5 different time points (20 days total)	Game Lacrosse: ~1050 kcal Basketball: ~1600 kcal Practice Lacrosse: ~980 kcal Basketball: ~1150 kcal Conditioning Lacrosse: ~800 kcal Basketball: ~1150 kcal Lacrosse: ~625 kcal	Game Lacrosse: ~2850 kcal·day^−1^ Basketball: ~3500 kcal·day^−1^ Practice Lacrosse: ~2700 kcal·day^−1^ Basketball: ~3000 kcal·day^−1^ Conditioning Lacrosse: ~2550 kcal·day^−1^ Basketball: ~3050 kcal·day^−1^ Off day Lacrosse: ~2400 kcal·day^−1^ Basketball: ~2480 kcal·day^−1^	Game Lacrosse: 1.85 Basketball: 2.2 Practice Lacrosse: 1.8 Basketball: 1.9 Conditioning Lacrosse: 1.7 Basketball: 1.9 Off day Lacrosse: 1.58 Basketball: 1.55
Zanders 2021 [39]	NCAA DII basketball (*n* = 13; height: 173 ± 13.6 cm; body mass: 74.6 ± 9.1 kg; body fat %: 27.1 ± 3.2%)	4 consecutive days at 5 different time points (20 days total)	Phase 1: 1196 ± 296 kcal Phase 2: 1252 ± 157 kcal Phase 3: 1028 ± 157 kcal Phase 4: 819 ± 160 kcal Phase 5: 969 ± 362 kcal	Phase 1: 3065 ± 361 kcal·day^−1^ Phase 2: 2866 ± 363 kcal·day^−1^ Phase 3: 2850 ± 159 kcal·day^−1^ Phase 4: 2674 ± 216 kcal·day^−1^ Phase 5: 2806 ± 419 kcal·day^−1^	Phase 1: 1.75 ± 0.27 Phase 2: 1.63 ± 0.22 Phase 3: 1.62 ± 0.15 Phase 4: 1.52 ± 0.17 Phase 5: 1.59 ± 0.23
Silva 2017 [87]	Elite basketball, handball, volleyball, triathlon, and swimming (*n* = 18; height: 172.4 ± 5.6 cm; body mass: 62.3 ± 7.4 kg; fat mass: 14.3 ± 2.7 kg)	1 day at 2 different time points (2 days total)	Beginning of the season: 1297 ± 498 kcal Main stage of the competition: 1670 ± 301 kcal	Beginning of the season: 3126 ± 520 kcal·day^−1^ Main stage of the competition: 3549 ± 317 kcal·day^−1^	N/A
Silva 2013 [88]	Elite junior basketball (*n* = 7; height 173.1 ± 3.3 cm; body mass: 64.0 ± 5.4 kg; body fat%: 20.0 ± 4.6%)	7-day monitoring period In season	2103 ± 272 kcal	3493 ± 242 kcal·day^−1^	2.6 ± 0.3
Other					
Torres-McGehee 2020 [20]	NCAA DI equestrian, soccer, beach volleyball, softball, volleyball, ballet (*n* = 121; height: 168.9 ± 7.7 cm; body mass: 63.6 ± 9.3 kg; fat-free mass: 48.4 ± 4.9 kg; body fat %: 26.1 ± 5.4)	7-day monitoring period	Total: 825.8 ± 350.3 kcal Equestrian: 403 ± 62 kcal Beach Volleyball: 1109 ± 158 kcal Softball: 811 ± 131 kcal Volleyball: 838 ± 78 kcal/day Ballet: 811 ± 408 kcal Soccer: 1187 ± 40 kcal	Total: 2428 ± 145 kcal·day^−1^ Equestrian: 2389 ± 117 kcal·day^−1^ Beach Volleyball: 2447 ± 86 kcal·day^−1^ Softball: 2550 ± 172 kcal/day Volleyball: 2357 ± 114 kcal·day^−1^ Ballet: 2468 ± 127 kcal·day^−1^ Soccer: 2468 ± 61 kcal·day^−1^	N/A
Fraczek 2019 [89]	Elite speed skating, cross-country skiing, mountain biking, volleyball, downhill skiing, middle-distance running, kayaking (*n* = 15, height: 172.5 ± 6.2 cm; body mass: 63.7 ± 5.2; fat-free mass: 50.5 ± 4.4 kg; body fat%: 21.2 ± 5.2%)	7-day monitoring period	N/A	Accelerometer: 2289 ± 286 kcal·day^−1^ Relative: 35.9 kcal·kg·day^−1^ Self-completed questionnaire: 3156 ± 620 kcal·day^−1^ Relative: 49.5 kcal·kg·day^−1^	1.75–2.0
Hill 2002 [90]	Elite lightweight rowing (*n* = 7; height: 168.8 ± 4.7 cm; body mass: 60.9 ± 2.3 kg; body fat %: 22.8 ± 5.1%)	14-day monitoring period	N/A	3957 ± 1219 kcal·day^−1^	N/A
Woodruff 2013 [41]	Inter-university volleyball (*n* = 10; height: 177 ± 5 cm; body mass: 75 ± 9.7 kg; body fat percent: 25.2 ± 6.9%	7-day monitoring period	Starters: 392–892 kcal Reserve: 416–734 kcal	3479 ± 604 kcal·day^−1^	N/A

kcal·day^−1^ = kilocalories per day; kcal·kg·day^−1^ = kilocalories per kilogram of body mass per day; kg = kilograms; cm = centimeter; yrs. = years; NCAA = National Collegiate Athletics Association; DI = Division I; DII = Division II; DIII = Division III. PAL = Total daily energy expenditure/resting metabolic rate.

**Table 4:** A summary of validated screening tools used to identify those at risk for low energy availability, eating disorders, female athlete triad, or body dissatisfaction.

Author Year	Name of Tool/Metric	Primary Focus	Direct or Indirect	Target Population
Low Energy Availability				
Loucks 1994, 2011 [6,27]	Energy Availability Assessment	Energy availability	Direct	All athletes
Melin 2014 [12]	Low Energy Availability in Females Questionnaire (LEAF-Q)	Identify those at risk of LEA	Indirect	Adult female athletes
Slater 2016 [130]	Low Energy Availability Amongst New Zealand Athletes (LEANZA) questionnaire	Identify those at risk of LEA	Indirect	Adult female athletes
Heikura 2021 [131]	Lab markers (i.e., T3, T4, LH, hepcidin, Testosterone, etc.)	Identify biomarkers that may indicate risk of LEA	Indirect	Female and male athletes
Staal 2018 [23]	Resting Metabolic Rate Ratio	Identify those at risk of LEA via suppressed metabolic rate	Indirect	Female ballet dancers
Eating Disorders				
Black 2003 [132]	The Bulimia Test-Revised	Screening test designed to assess bulimia-type characteristics	Indirect	Female athletes
Garner 1982 [133]	Eating Attitudes Test (EAT)	Screening tool for anorexia nervosa	Indirect	Adult females
Berg 2012 [134]	Eating Disorder Examination Questionnaire	Self-reported questionnaire for the assessment and diagnoses of the DSM-IV eating disorders	Indirect	Adult females
Garner 2004 [135]	Eating Disorder Inventory (EDI-3)	Self-reported measure for identifying eating disorder patterns and associated psychological constructs	Indirect	Valid in individuals aged 13 to 53 years for identifying disordered eating patterns and has high reliability (Cronbach a mean = 0.94, range = 0.90–0.97)
Kennedy 2021 [136]	Disordered Eating Screening Tool for Athletes (DESA-6)	Screening tool for disordered eating	Indirect	Adolescent athletes
Martinsen 2014 [137]	Brief Eating Disorder in Athletes Questionnaire (BEDA-Q)	Brief questionnaire able to discriminate between female elite athletes with and without an eating disorder	Indirect	High school female athletes
McNulty 2001 [138]	Female Athlete Screening Tool (FAST)	Screening tool to identify eating disorders	Indirect	Collegiate female athletes
Nagel 2000 [139]	Athletic Milieu Direct Questionnaire (AMDQ) version 2	Screening tool to identify eating disorders	Indirect	Female athletes
Stice 2004 [140]	Eating Disorder Diagnostic Scale	A brief self-report measure for diagnosing anorexia nervosa, bulimia nervosa, and binge eating disorder	Indirect	Adolescent, collegiate, and adult females
Female Athlete Triad				
Otis 1997 [141]	Menstrual Cycle	Used to assess delayed menarche, menstrual irregularities or amenorrhea	Direct	Female athletes
Otis 1997 [141]	Bone Mineral Density	Low BMC or BMD * is defined as a BMC or areal BMD Z-score that is ≤−2.0, adjusted for age, gender and body size, as appropriate. [142] * American College of Sports Medicine (ACSM) defines low BMC or BMD as a Z-score that is less than −1.0 in female athletes in weight-bearing sports	Direct	Multiple populations
Otis 1997 [141]	Eating Disorder (see above examples: FAST. AMDQ, DESA-6, EAT, EDI-3, BEDA-Q)	See above examples	Indirect	
Body Image/Dissatisfaction				
Orbach 1998 [143]	Body Image Investment Scale	Identify those at risk for body image issues	Indirect	Boys and girls (age: 13–19 years) 3–19 years
Garner 2004 [135]	Eating Disorder Inventory-3 subscale c: body dissatisfaction	Identify risk factors for eating disorder associated with body dissatisfaction	Indirect	Valid in individuals aged 13 to 53 years for identifying disordered eating patterns and has high reliability (Cronbach a mean = 0.94, range = 0.90–0.97)
Sandoz 2013 [144] Lucena-Santos 2017 [145]	The Body Image-Acceptance and Action Questionnaire	Evaluates body image flexibility and dissatisfaction	Indirect	Females
Cash 1995 [146]	Body-Image Ideals Questionnaire	Attitudinal body-image assessment that considers physical attributes	Indirect	Female college students
Cash 2004 [147]	Body Image Disturbance Questionnaire	Assessed body image disturbance	Indirect	Male and female college students
Kong 2013 [7]	Figure Rating Scale	Identify body satisfaction and views on body shape	Indirect	Female athletes
Cooper 1987 [148] Goltz 2013 [149]	Body Shape Questionnaire	Identify concerns associated with body image	Indirect	Young men and women, athletes and non-athletes

LH = Luteinizing hormone, T3 = Triiodothyronine; T4 = Thyroxine.

## Data Availability

Data sharing not applicable: No new data were created or analyzed in this study.
